# The Impact of Chronic Stress on Treatment Outcomes of Cancer Patients with Divergent Survival Rates: A Systematic Review

**DOI:** 10.3390/ijms27020686

**Published:** 2026-01-09

**Authors:** Katarzyna Herbetko, Justyna Kaczor, Adam Sołtyk, Monika Kisielewska, Marcel Opęchowski, Aleksandra Sztuder, Julita Kulbacka

**Affiliations:** 1 Faculty of Medicine, Wroclaw Medical University, Pasteura 1, 50-367 Wroclaw, Poland; katarzyna.herbetko@student.umw.edu.pl (K.H.); justyna.kaczor@student.umw.edu.pl (J.K.); adam.soltyk@student.umw.edu.pl (A.S.); monika.kisielewska@student.umw.edu.pl (M.K.); marcel.opechowski@student.umw.edu.pl (M.O.); 2 Student Research Group No. SKN148, Faculty of Pharmacy, Wroclaw Medical University, 50-556 Wroclaw, Poland; 3 Department of Oncology, Wroclaw Medical University, pl. L. Hirszfelda 12, 53-413 Wroclaw, Poland; aleksandra.sztuder@umw.edu.pl; 4 Department of Radiotherapy, Lower Silesian Oncology, Pulmonology and Haematology Center, pl. L. Hirszfelda 12, 53-413 Wroclaw, Poland; 5 Department of Molecular and Cellular Biology, Faculty of Pharmacy, Wroclaw Medical University, Borowska 211a, 50-556 Wroclaw, Poland; 6 Department of Immunology and Bioelectrochemistry, State Research Institute Centre for Innovative Medicine, Santariškių g. 5, LT-08406 Vilnius, Lithuania

**Keywords:** stress, chronic stress, cancer, psychological care, cancer survival rate

## Abstract

This systematic review investigates the impact of chronic stress on treatment outcomes among cancer patients with divergent survival rates, focusing on breast, prostate, pancreatic, and ovarian cancers. The analysis explores how chronic stress influences molecular pathways and tumor progression while comparing cancers with five-year survival rates above and below 50%. A comprehensive literature search was conducted in PubMed and Scopus for studies published between 2014 and 2025 using combinations of keywords related to “chronic stress,” “psychological stress,” “psychotherapy,” and selected cancer types. All studies met the inclusion criteria according to the PRISMA 2020 guidelines. Evidence suggests that chronic stress is associated with the activation of neuroendocrine and immune mechanisms, including β-adrenergic and glucocorticoid signaling. These multifactorial processes are associated with disease progression and survival, particularly in pancreatic and ovarian cancers; however, these links remain primarily associative rather than causative. Conversely, psychotherapeutic interventions alleviate stress-related biological responses, improve quality of life, and may indirectly enhance therapeutic efficacy. By structuring the evidence around cancers with higher versus lower five-year survival, our review provides a survival informed synthesis of cancer type specific stress biology and stress-mitigating interventions, highlighting potentially targetable pathways and clear evidence gaps for future trials. The findings underscore the need to integrate psychological care into oncological practice to improve overall outcomes.

## 1. Introduction

Stress is defined as a phenomenon that occurs when environmental demands challenge an individual’s adaptive capacity. In the oncologic field, it can be described as a multicausal, multilevel experience that involves the social, emotional, and spiritual dimensions of being, interfering with the ability and willingness to confront the illness [1].

Chronic stress can be one of the causes of tumorigenesis, as the analysis of retrospective studies conducted in this field reveals that cancer patients outweigh controls in terms of quantity when it comes to stressful experiences [2]. What is more, it can increase the metastasis and recurrence probability [3], as well as contribute to cancer-related post-traumatic stress disorder (PTSD) and depression in already-diagnosed patients [4,5].

**Figure 1:**
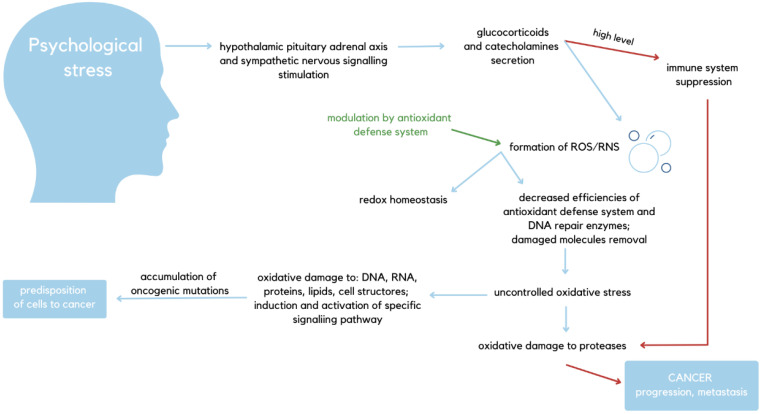
Schematic visualization of psychological stress’s impact on homeostasis regarding its role in carcinogenesis. Psychological stress stimulates both sympathetic nervous system signaling and HPA axis signaling. These result in the secretion of glucocorticoids and catecholamines, which in turn suppress the immune system and stimulate ROS and RNS formation, thus causing oxidative damage and supporting cancer progression [5,26,27,28,29,30,31,32,33].

The interaction between chronic stress, immune reactivity, and tumor development makes the described factor able to worsen the physical condition of the patient [6]. In addition to psychosocial and neuroendocrine mechanisms, nutritional deficiencies, particularly in vitamins B and D, selenium, magnesium, and zinc, can exacerbate chronic stress by impairing antioxidant defense, neurotransmitter synthesis, and immune balance. These micronutrients regulate oxidative stress and hormonal signaling, influencing both stress adaptation and cancer progression [7,8]. In response to chronic stress, the human organism activates various pathways that broadly affect the physical manifestation of cancer. The central sympathetic nervous system and the hypothalamus–pituitary–adrenal axis are stimulated, leading to the release of catecholamines and glucocorticoids [9]. Those mediators play key roles in the chronic stress clinical picture and are able to interfere with some types of cancers. For example, catecholamines were proven to augment carcinogenic properties of colon, prostate, ovary, and breast tumors [10]. Glucocorticoids, on the other hand, constitute immunosuppressive agents that impair cancer-related defense pathways. As various immune cells directly influence tumor formation and growth, as well as metastasis, the fact that psychological factors impact the immune system in many ways makes them significant in the tumor development process [11]. While chronic stress has been shown to activate neuroendocrine and immune mechanisms, these biological responses are modulated by numerous factors, including genetic background, comorbidities, nutritional status, and treatment type. Therefore, the relationship between psychological stress and specific hormonal or cytokine changes should be interpreted as associative rather than strictly causal. Not only does chronic stress impair adhesion molecule expression and chemotaxis, but it also reduces NK (Natural Killer) cytotoxic capacity. Moreover, under stressful conditions, the neuropeptide substance P, released from sensory nerve endings, acts as a mediator linking the nervous and immune systems. It stimulates macrophages to increase the production of pro-inflammatory cytokines such as IL-1 and TNF-α, thereby contributing to inflammation and immune dysregulation. Concurrently, stress suppresses T-cell mobilization through β_2_-adrenergic receptor pathways, collectively impairing anti-tumor immune responses [12]. Furthermore, stress modulates brain-blood barrier permeability via acute-phase proteins, such as IL-1α. An increase in psychoactive substances and drug inflow to the brain, thus making the organism more sensitive towards drugs as well as daily used substances [13] (Figure 1). What is more, chronic stress activates β-adrenergic receptors, consequently leading to the stimulation of signaling pathways that activate essential oncogenes that lead to tumor development [14].

At a systemic level, chronic stress acts as a biological accelerator, linking neuroendocrine dysregulation with molecular processes of both accelerated aging and tumorigenesis. Prolonged activation of the hypothalamic–pituitary–adrenal (HPA) axis and the sympathetic nervous system triggers persistent release of glucocorticoids and catecholamines, which, in turn, disrupt immune surveillance, promote inflammation, and enhance oxidative and nitrosative stress [15,16]. These stress mediators contribute to telomere shortening, DNA damage, and mitochondrial dysfunction, hallmarks of biological aging that also facilitate malignant transformation and tumor progression. Moreover, chronic inflammation and stress-induced hormonal signaling (e.g., β-adrenergic and glucocorticoid receptor pathways) converge on oncogenic transcription factors such as Nuclear Factor Kappa-light-chain-enhancer of Activated B Cells (NF-κB) and Signal Transducer and Activator of Transcription 3 (STAT3), creating a microenvironment conducive to both cellular senescence and cancer growth [17]. Figure 1 schematically illustrates this broad psychobiological pathway through which stress exacerbates cancer development before the disease-specific mechanisms discussed in later sections.

Differences in the biological and psychosocial context of specific cancers suggest that stress may not exert uniform effects across all malignancies. Distinct cancer types vary in their biological stress responsivity due to differences in tissue origin, hormonal regulation, immune microenvironment, and treatment intensity. For example, hormone-dependent cancers such as breast and prostate neoplasms show pronounced sensitivity to adrenergic and glucocorticoid signaling, while pancreatic and ovarian cancers display high inflammatory and cytokine-driven stress pathways. Moreover, perceived stress and coping differ across diagnoses: cancers with poorer prognoses (e.g., pancreatic, ovarian) are associated with higher emotional burden, depressive symptoms, and systemic inflammation, whereas cancers with higher survival rates (e.g., breast, prostate) often involve chronic anxiety but greater psychological resilience. Differences in therapeutic burden, including aggressive chemotherapy, surgical morbidity, or hormonal therapy, further alter neuroendocrine and immune pathways [18,19]. Together, these disease-specific biological and psychosocial factors justify a granulated review that compares stress biology and outcomes across cancer types. However, it is equally important to acknowledge the influence of individual variability in psychological stress levels, which may arise from personal history, coping styles, socioeconomic context, or social support. Such interindividual differences can modulate stress-related biological responses and partially obscure cancer-type-specific patterns, making the delineation of universal mechanisms particularly challenging [20]. This multidimensionality underscores the complexity of linking stress biology to cancer outcomes and highlights the need for nuanced, disease-specific analyses. For instance, cancer patients with low socioeconomic status often struggle to cope with cancer-related stress, which heightens their vulnerability to mental health disorders. Social inequalities influence both physical and psychological well-being, potentially through differences in health behaviors and health knowledge, which highlights the need for personalized survivorship care plans [21]. The close interaction between oncology and mental health contributes to a high prevalence of psychological disorders among cancer patients, further stressing the need for integrated care approaches. Evidence supports the effectiveness of culturally informed collaborative care models that combine mental and physical health interventions to enhance patients’ overall well-being and resilience. Incorporating such holistic, culturally sensitive frameworks into oncological practice can help reduce psychological distress and stigma while improving treatment outcomes and quality of life (QOL) for cancer patients [22]. It must also be noted that variations in cytokine or hormone levels in cancer patients arise from a combination of psychosocial, physiological, pharmacological, and disease-related factors. Hence, attributing these changes only to psychological stress generalizes the complex interplay of biological factors.

Although chronic stress’s influence on various oncological patients has been examined thoroughly, the relationship between the level of stress that accompanies certain types of cancer and patients’ survival should be examined more carefully and with regard to its molecular background. For this reason, we divided neoplastic diseases into two groups, based on 5-year relative survival rates for all stages and races combined. We considered survival rates above 50% high and those below 50% low. According to the National Center for Health Statistics (NCHS), cancers with a 5-year survival rate above 50% include breast and prostate cancer, whereas those under 50% include both pancreatic and ovarian cancer [23]. The aim of analyzing the data was to determine how chronic stress influences tumor formation and progression, as well as to verify the hypothesis that claims psychotherapy is an important potential aspect of oncological treatment. Even though each patient constitutes an inimitable individual and therefore reacts differently to the diagnosis of cancer, general tendencies suggest that being diagnosed with an illness that is generally believed to be fatal increases patients’ psychological stress level, thus decreasing survival chances [24]. On the other hand, when it comes to neoplastic diseases classified as the first group, optimistic prognoses make patients hope for a quick recovery, thereby contributing to a lower morbidity rate [25]. Psychological factors, therefore, seem to be of great significance for cancer survival. However, all conclusions should be interpreted with caution. By examining cancer-type-specific stress biology, this review aims to clarify how distinct neuroendocrine and immune responses may contribute to divergent survival outcomes and therapeutic sensitivities.

Our review provides conceptual novelty by implementing a survival-stratified framework that categorizes malignancies based on five-year relative survival rates above and below 50%. This integrated mechanistic perspective allows us to separate pathways consistently linked to metastasis and therapy resistance from those dominated by inflammatory and cytokine-driven biology. By aligning these specific mechanisms with risk-adapted psychosocial interventions, we provide a more granular synthesis than is typically found in broader psycho-oncological literature.

## 2. Methods

### 2.1. Study Design

This systematic review was designed and reported in accordance with the PRISMA 2020 guidelines [34]. The study protocol was registered on the Open Science Framework (OSF), under the DOI: https://doi.org/10.17605/OSF.IO/5ZF2U. The review aimed to synthesize current evidence on the impact of chronic stress on treatment outcomes and survival across cancer types with divergent five-year survival rates.

### 2.2. Research Questions

The review addressed the following questions:

(1) How does chronic stress influence cancer progression and treatment outcomes?
(2) Do these effects differ between cancers with high versus low survival rates?
(3) What molecular and psychological mechanisms mediate these relationships?
(4) Which psychotherapeutic interventions have been shown to mitigate stress-related effects?

### 2.3. Information Sources and Search Strategy

A systematic search was conducted in PubMed and Scopus databases for articles published between January 2014 and April 2025. The search combined controlled vocabulary (MeSH) and free-text terms related to chronic stress, psychological stress, psychotherapy, and specific cancers (breast, prostate, pancreatic, ovarian). Example PubMed search string:

(“chronic stress” OR “psychological stress” OR “stress response”) AND (“breast cancer” OR “prostate cancer” OR “pancreatic cancer” OR “ovarian cancer”) AND (“therapy” OR “treatment outcome” OR “survival”).

The reference lists of included papers and relevant reviews were also manually screened to identify additional studies.

### 2.4. Eligibility Criteria

Studies were included if they met all the following criteria:

- Peer-reviewed articles available in English;
- Published between 2014 and 2025;
- Examined associations between chronic/psychological stress and treatment outcomes, survival, or related molecular mechanisms in the selected cancer types;
- Employed quantitative, qualitative, or mixed-methods design with adequate methodology.

Exclusion criteria:

- Conference abstracts, preprints, letters, or commentaries;
- Studies lacking defined outcome measures or psychological variables;
- Animal studies without translational relevance.

### 2.5. Study Selection

**Figure 2:**
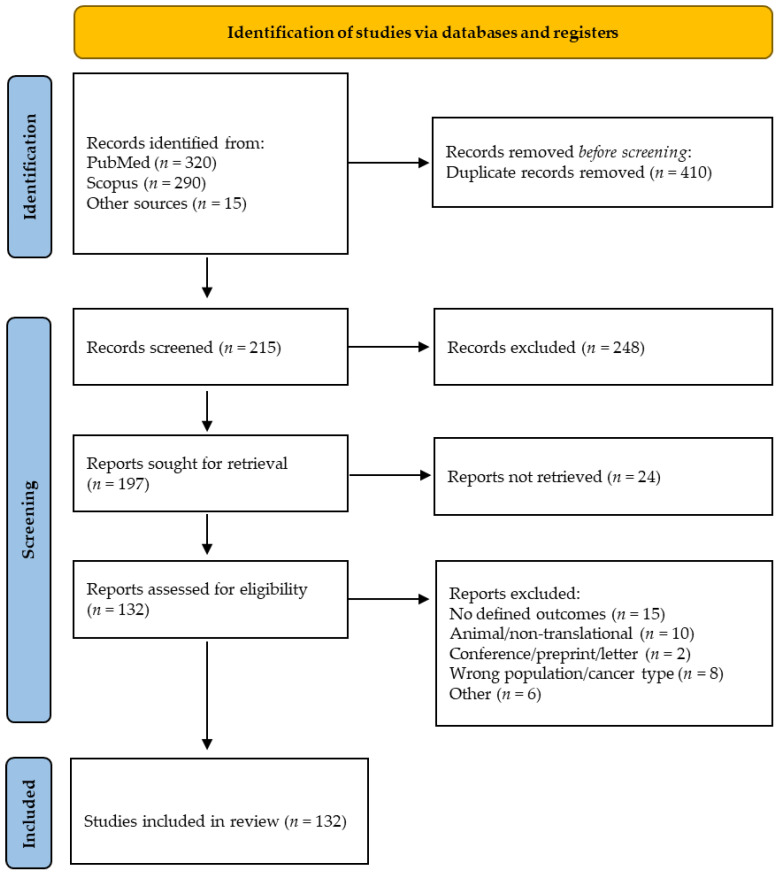
PRISMA 2020 flow diagram showing identification, screening, and inclusion of studies for the systematic review.

All retrieved records were imported into EndNote X9 to remove duplicates. Two reviewers independently screened titles and abstracts for relevance. Full texts of potentially eligible papers were evaluated by both reviewers, and discrepancies were resolved by consensus or with a third reviewer. The PRISMA flow diagram (Figure 2) illustrates the selection process.

### 2.6. Data Extraction and Management

Data were extracted using a standardized Excel form that captured

- Author, year, country;
- Cancer type and sample size;
- Study design and main psychological variables;
- Molecular mechanisms explored;
- Primary outcomes (treatment response, progression, survival, quality of life);
- Key conclusions.

### 2.7. Risk of Bias Assessment

The methodological quality of observational studies was assessed using the Joanna Briggs Institute (JBI) critical appraisal checklist for cross-sectional and cohort designs. Each study was rated as low, moderate, or high risk of bias [35].

### 2.8. Data Synthesis

Given the heterogeneity of study designs and outcomes, findings were synthesized narratively using a thematic approach, grouping results by cancer type and mechanistic pathway. Quantitative pooling was not feasible. Key pathways were visualized in conceptual diagrams to facilitate interpretation.

### 2.9. Limitations

Although every effort was made to ensure comprehensive coverage, publication bias and heterogeneity in psychological stress measures limit the generalizability of conclusions. Nevertheless, adherence to PRISMA 2020 standards enhances the transparency and reproducibility of this review.

## 3. Results and Discussion

### 3.1. High Survival Rate

Globally, decreased QOL may serve as a marker of disease progression and symptom burden rather than an independent prognostic factor for mortality. However, the relationship between psychological stress and specific hormonal or cytokine changes is interpreted as associative. Biological effects are supported by the observation that stress mediators, such as catecholamines and glucocorticoids, disrupt immune surveillance and promote inflammation. It is reasonable that as disease severity increases, symptom burden and functional limitations worsen, thereby reducing QOL. Thus, decreased QOL may serve as a marker of disease progression rather than an independent prognostic factor for mortality. Nevertheless, evidence suggests that baseline (pretreatment) QOL remains a useful integrative indicator of patients’ physical and psychological status and can provide complementary prognostic information when considered alongside clinical variables [36].

#### 3.1.1. Breast Cancer

Breast cancer (BC) is diagnosed in one in eight women in the Western world. It thus constitutes the most common non-cutaneous malignancy in women, with a 90% 5-year relative survival rate and an 84% 10-year relative survival rate [37,38]. Environmental factors are generally estimated to constitute a cause of 70% of malignant tumors; however, when it comes to BC, that number ranges from 90 to 95% [39]. A significant percentage of BC patients experience varied psychological symptoms from the moment the diagnosis is stated, through the therapy, and after it comes to an end. Acceptance of difficult situations, coping with possible side effects of the treatment, as well as facing a permanent sense of uncertainty that escalates during every follow-up examination, constitute the factors that are able to cause psychological symptoms. This can, in turn, result in the occurrence of long-term mood disorders, such as depression and PTSD [4,40]. In recent studies, women diagnosed with metastatic BC reported several stress factors during diagnosis, including difficulties communicating with doctors and a perceived delay in receiving their diagnosis. Emotional distress, worries about family, and reflections on why the cancer developed further contributed to their psychological strain [41]. The stress experienced by BC survivors encompassed psychological distress, physical pain, financial strain, lifestyle changes, and the burden of information overload [42]. In other studies, patients commonly reported moderate to severe distress. Major stress factors included concerns related to work, home care, and family responsibilities, as well as feelings of isolation, lack of support, and issues with body image. Additionally, physical symptoms such as fatigue, appetite loss, and sleep disturbances, along with social and interpersonal difficulties, further contributed to their overall psychological burden [43].

The main cause of death in breast cancer patients is metastases, which can be accelerated by chronic stress via β-adrenergic receptors (βARs) activation [44,45]. For instance, adrenaline increases phosphorylation of p38 mitogen-activated protein kinase (MAPK) in breast cancer cells. It enhances tumor growth, which suggests that the activation of adrenaline-induced p38 MAPK signaling pathway augments the malignancy of breast cancer in depressive disorders [46]. The role of the βAR pathway was demonstrated in MDA-MB-231 breast cancer cells, where β2AR signaling facilitates invasion in vitro [44]. However, the described dependencies are not obvious under in vivo conditions; thus, relevant research was conducted to investigate the role of β2AR signaling in breast tumor cell metastasis within a living organism. In this respect, both MDA-MB-231 breast cancer cells, deficient in β2AR, and low-metastatic Michigan Cancer Foundation-7 (MCF-7) BC cells with β2AR overexpression were generated. As a result, β2AR knockdown in breast tumor cells reduced the impact of stress on metastasis in vivo. As envisaged, overexpression of β2AR encouraged an invasive phenotype. Therefore, the significant role of the described signaling path was emphasized, suggesting that blocking β2AR on breast tumor cells could enable us to control metastatic progression [44]. A crucial role in cancer progression is also played by angiogenesis [29]. Chronic stress related to social isolation significantly inhibits the expression of peroxisome proliferator-activated receptor gamma (PPARγ), thereby elevating vascular endothelial growth factor/fibroblast growth factor 2 (VEGF/FGF2) and, consequently, promoting breast cancer angiogenesis in vivo [14,29].

**Figure 3:**
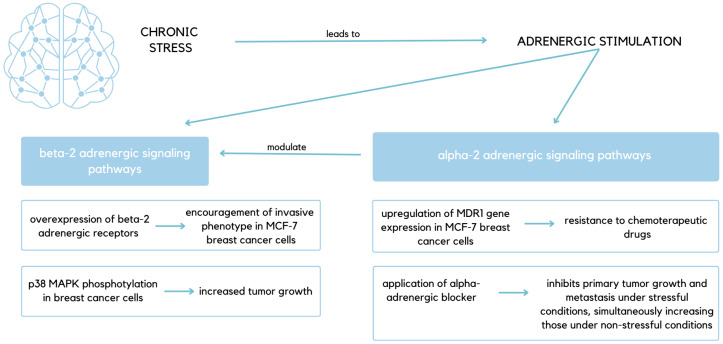
Schematic visualization of chronic stress’s impact on adrenergic signaling pathways. Presented dependencies underline the importance of stress-induced adrenergic stimulation, which results in phenomena such as increased resistance to chemotherapeutic drugs and increased tumor growth [44,46,48,49].

Apart from βAR, catecholamines also signal through α-adrenergic receptors (αAR), and this signaling has been shown to promote breast cancer in both in vitro and in vivo models. The complicity of that discovery lies in the antagonism of αAR, which may increase catecholamine levels. These elevated levels may increase β-adrenergic signaling, as presynaptic α2AR mediates autoinhibition of sympathetic transmission. For this reason, a mouse model with MDS-MB-231 breast tumor cells was used to examine the effect of α-adrenergic blockade on breast cancer progression under stress and non-stress conditions. The group of mice affected by stress exhibited increased primary tumor growth and metastasis. In their case, administering phentolamine, a non-selective α-adrenergic blocker, specifically inhibited these effects. Conversely, under non-stress conditions, the application of that blocker increased both primary tumor growth and metastasis to distant tissues. When it comes to selective α-blockers, efaroxan, a selective α2-adrenergic blocker, had compatible effects on tumor and metastasis under non-stressful conditions. In contrast, prazosin, a selective α1-adrenergic blocker, did not increase either primary tumor growth or metastases. The obtained results prove that α2-adrenergic signaling autoregulates by inhibiting sympathetic catecholamine release and simultaneously modulating the effects of β-adrenergic signaling (Figure 3) [47,48].

Adrenergic stimulation has been shown to upregulate Multidrug Resistance Protein 1 (MDR1) gene expression in MCF-7 BC cells via α2AR, and this process can be inhibited by MDR1-siRNA pretreatment. In this way, the stimulation augments P-glycoprotein function, therefore conferring MCF-7 cells resistance to paclitaxel. In a mouse model, administration of adrenaline significantly increases MDR1 mRNA levels. Regarding MCF-7 cancer cells injected subcutaneously into Severe Combined Immunodeficiency (SCID) mice, restraint stress increased MDR1 gene expression, thereby conferring resistance to doxorubicin. A described cascade of events can be blocked with an α2-adrenergic inhibitor administration, yohimbine [49]. As described, research demonstrates that BC can develop resistance to chemotherapeutic drugs under conditions of chronic stress that leads to MDR1 gene overexpression resulting from adrenergic stimulation (Figure 3) [45]. Moreover, chronic stress in patients with BC promotes the progression of cancer via activation of Tumor-Associated Macrophages/C-X-C Motif Chemokine Ligand 1 (TAM/CXCL1) signaling dependent on glucocorticoid–glucocorticoid receptor interactions [14]. Additionally, the role of CXCL1-C-X-C Motif Chemokine Receptor 2 (CXCR2) signaling has been emphasized when it comes to mobilizing MDCSs (myeloid-derived suppressor cells) into the lung to establish a pre-metastatic niche (PMN), thus facilitating metastasis [14]. Therefore, the role of the spleen in chronic stress-induced BC warrants further investigation.

#### 3.1.2. Prostate Cancer

Prostate cancer (PCa) is the most common cancer among men and the fifth leading cause of cancer deaths, with an estimated 1.6 million new cases and 366,000 deaths in 2015 worldwide. Considering the cancer stages, the survival rate varies by stage from 99.3% for the locoregional stage to 32.3% with distant metastasis [50]. Chronic stress among prostate cancer patients is substantial. Aspects of PCa diagnosis and treatment that contribute to the highest emotional stress are the initial diagnosis, its unknown outcome, the possibility of death, loss of QOL, or loss of a partner [51]. PCa patients also have a relatively high prevalence of depression and anxiety [52]. In recent studies, perceived stress was identified as a key determinant of both functional and emotional outcomes among PCa survivors. High stress levels negatively influenced overall well-being, while psychological resilience emerged as a crucial protective factor that mitigated the detrimental effects of stress and threats to masculine identity, thereby supporting better emotional adjustment and recovery [53]. Moreover, adjuvant androgen deprivation therapy (ADT) was correlated with higher distress as distinct from patients with follow-up only, as it resulted in depression, worse QOL, and disturbed self-image [54]. Additionally, some studies demonstrate that PCa patients may experience specific anxiety related to PSA (PSA anxiety), and higher PSA values may conceivably be associated with elevated stress levels [55]. The studies also show disparities in stress among patients. Research findings present substantially higher stress levels in African American men in comparison to non-African Americans [56]. The level of distress also varies by age. Aging is associated with lower levels of distress and anxiety, which indicates that older PCa patients manage the disease better than younger patients. However, it should be taken into consideration that older patients report more depressive symptoms [57]. It was also assessed that the higher distress is related to increased cancer mortality, including prostate cancer; therefore, taking cognizance of the psychological state of PCa patients is crucial during treatment [58].

**Figure 4:**
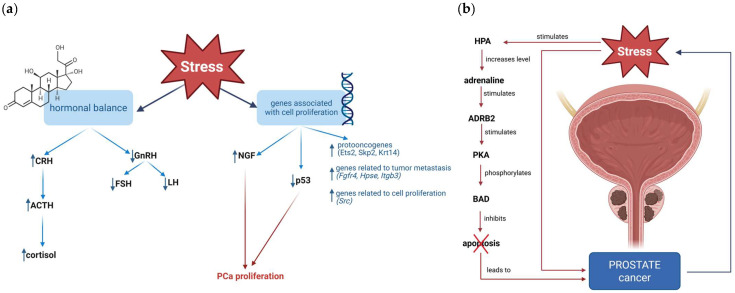
(**a**) Schematic visualization of how stress influences hormonal balance and expression of genes associated with cell proliferation and metastases [59,61]. (**b**) Schematic visualization of the stress-induced adrenaline/Adrenergic Receptor Beta (ADRB)/Protein Kinase A (PKA)/Bcl-2-Associated Death Promoter (BAD) pathway and its connection with prostate cancer. Chronic stress stimulates the HPA, which increases adrenaline levels. The adrenaline-stimulated β2-adrenergic receptor (ADRB2), which in turn activates PKA, phosphorylates BAD, thereby inhibiting apoptosis and promoting prostate cancer [62,63,64]. Created in BioRender. Kulbacka, J. (2026) https://BioRender.com/8dy2o2q and https://BioRender.com/vgd493r.

Chronic stress promotes prostate cancer progression by activating the HPA axis, which stimulates the secretion of corticotropin-releasing hormone (CRH) from the paraventricular nucleus of the hypothalamus (PVH). CRH consecutively leads to adrenocorticotropic hormone (ACTH) secretion by the anterior pituitary, which, in turn, induces the adrenal cortex to release cortisol. In addition to activating the HPA axis, chronic stress also inhibits the hypothalamic–pituitary–gonadal (HPG) axis, leading to decreased GnRH secretion and, subsequently, reduced LH and FSH levels. GnRH agonists have been used in prostate cancer treatment as they suppress the proliferation of reproductive cancer cells, thus GnRH may have an antineoplastic activity. The HPA axis’s stress-induced activation was confirmed in studies examining male rats exposed to both acute and repeated restraint stress. Increased neuronal activity in the PVH was demonstrated by elevated Fos expression in immunohistochemically stained brain tissue. Furthermore, the study established that stress affects the expression of genes associated with cell proliferation and metastasis in the prostate. PCR analysis of prostate tissues showed increased expression of numerous cancer-related genes, including proto-oncogenes such as Ets2 (V-ets erythroblastosis virus E26 oncogene homolog 2) and Skp2 (S-phase kinase-associated protein 2, p45), as well as the prostate cancer marker Krt14 (Keratin 14). In addition, stress exposure led to upregulation of genes associated with tumor metastasis. Acute stress influenced the expression of fibroblast growth factor receptor 4 (Fgfr4), heparanase (Hpse), and integrin beta 3 (Itgb3), whereas repeated stress affected multiple gene expression levels, including Src, which stimulates cell proliferation. The expression levels of most genes returned to normal after recovering from stress, except for Chemokine (C-C motif) ligand 7 and Plasminogen activator, urokinase receptor. Repeated stress also reduced p53 expression, which remained downregulated even after recovery. The majority of the above-mentioned genes are regulated by cortisol; thus, alterations in their expression may be directly induced by increased levels [59]. Chronic stress can also lead to histological changes in the prostate, which has been proven in research during which rats were exposed to restraint stress. After 14 days of restraint water-immersion stress, the proliferation of ventral lobes of the prostate was ascertained. In contrast, the changes were absent when stress was combined with the denervation of the peripheral sympathetic nerve. The results indicate that chronic stress may result in epithelial hyperplasia of the ventral prostate [60]. A subsequent study showed that nerve growth factor (NGF), a crucial prostatic mitogen, may be linked to prostate hyperplasia (Figure 4a). Stress and chemical sympathectomy independently increased NGF expression in the ventral lobes. The NGF level was higher in the group with combined restraint water-immersion stress and denervation than in the previous ones. These results imply that NGF may be related to the development of prostate disease [61].

Chronic stress increases adrenaline levels. Hence, stress activates the adrenaline/ADRB/PKA/BAD antiapoptotic signaling pathway, which accelerates prostate tumor progression. Adrenaline stimulates ADRB2, which induces BAD phosphorylation by PKA. BAD phosphorylation inhibits apoptosis and, in turn, leads to tumor development (Figure 4b). The role of stress in BAD phosphorylation was evaluated in research involving mice with prostate-restricted expression of the proto-oncogene c-MYC (Hi-Myc mice) and C42 xenografts in nude mice. Immobilization stress induced BAD phosphorylation, which inhibited apoptosis and resulted in enlarged prostatic intraepithelial neoplasia (PIN) and increased prostate weight overall. Moreover, stress elicited therapy resistance as it attenuated the effectiveness of androgen ablation therapy [64]. The other study additionally demonstrated that the adrenaline/ADRB/PKA/BAD pathway can alter drug combinations from synergy to antagonism [63]. The studies mentioned above imply that chronic stress, which is often experienced in prostate cancer patients, may increase tumor development and drug resistance.

**Figure 5:**
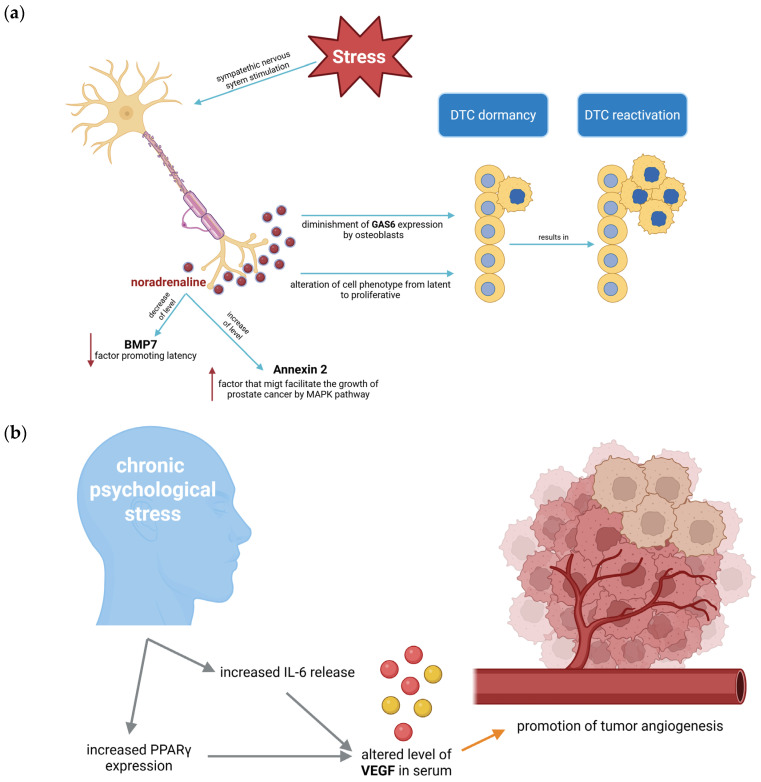
Schematic visualization of the role that stress-induced noradrenaline release plays in the process of DTC reactivation. (**a**) Stress stimulates the sympathetic nervous system, leading to the release of noradrenaline. Noradrenaline exhibits pleiotropic actions: it increases Annexin 2 levels and decreases BMP7 levels, diminishes GAS6 expression by osteoblasts, and alters cell phenotype in a proliferative direction [29,65]. (**b**) The visualization of how chronic stress impacts tumor angiogenesis by increasing the level of IL-6 that stimulates VEGF release [26,29,62,66]. Created in BioRender. Kulbacka, J. (2026) https://BioRender.com/42uh1yx and https://BioRender.com/bjbbw8o.

Noradrenaline, another stress-increased catecholamine, has been implicated as a crucial factor responsible for the relapse of metastatic prostate cancer. Noradrenaline reactivates disseminated tumor cells (DTCs) in the bone marrow and therefore affects PCa recurrence. In approximately 80% of cases of PCa, metastases occur in the bones. Disseminated tumor cells enter the bone marrow, where they can proliferate, become latent, or undergo apoptosis. Dormant DTCs may be reactivated after many years, resulting in relapse, for instance, by adrenergic neurotransmitters. Noradrenaline can induce re-entry into the cell cycle of tumor cells by diminishing growth arrest-specific-6 (GAS6) expression in osteoblasts, or it can directly alter the cell phenotype from latent to proliferative (Figure 5a). Moreover, noradrenaline may mobilize hemopoietic stem cells (HSC) from the bone marrow into the bloodstream. DTCs exhibit a phenotype similar to that of stem cells; thus, the signals that mobilize HSCs may also activate dormant PCa cells. Noradrenaline stimulation also increases adhesion protein Annexin 2 levels and decreases bone morphogenetic protein 7 (BMP7) levels, both of which promote tumor cell latency. These findings suggest that stress can mobilize latent DTCs by stimulating the sympathetic nervous system, leading to catecholamine release. Furthermore, stress may promote DTC activation by suppressing the immune system and releasing pro-inflammatory factors [65].

Chronic stress in prostate cancer patients, which is aggravated due to the diagnosis and treatment, leads to increased tumor progression, mortality rate, and the likelihood of relapse, and it can also provoke therapy resistance. All of the enumerated factors lower the survival rate among patients with PCa. Thus, patients should have easy access to psychological support before and during treatment.

### 3.2. Low Survival Rate

#### 3.2.1. Pancreatic Cancer

Diagnosis of cancer is always connected with a large amount of stress, which is inflicted on patients within a short time. Especially when it comes to one of the more deadly cancers, such as pancreatic cancer (PC), in a year from diagnosis. Pancreatic adenocarcinoma has a very poor prognosis; typically, after diagnosis, only 24% of people survive 1 year, and 9% live for 5 years [67,68]. Such a diagnosis is an enormous burden for patients, which leads to the fact that 40% of them are affected with psychological distress, and 30% of them report symptoms of clinical depression [69,70]. Numerous studies implicate that distress and other psychological symptoms are a demonstration of pancreatic cancer, while others show that stress is a contributing factor in its development. That can be associated with the fact that up to 50% of patients with pancreatic cancer admit to developing psychological symptoms up to 3 years before the start of the physical symptoms of pancreatic cancer [71]. Due to the current state of knowledge, the exact connections between stress and pancreatic cancer are not precisely known [72] is still a growing number of studies that examine mechanisms linking stress and pancreatic cancer.

Previous research has primarily focused on variable-centered approaches, demonstrating significant associations between psychological capital and death anxiety. However, limited attention has been given to the heterogeneity of these psychological constructs among patients with PC. Recent evidence using latent profile analysis has identified distinct psychological patterns, characterized by varying levels of psychological capital and death anxiety. Factors such as gender, age, place of residence, and cancer stage appear to influence these profiles. These findings highlight the importance of recognizing individual differences in psychological responses and underscore the need for tailored psychosocial interventions to enhance mental health and coping among pancreatic cancer patients [73]. Patients with pancreatic ductal adenocarcinoma (PDAC) tend to exhibit higher levels of depressive symptoms both at diagnosis and during follow-up compared to those with other pancreatic diseases. Conversely, individuals without PDAC report experiencing a greater number and severity of lifetime stressors. Overall, greater exposure to stressful life events, considering their frequency, intensity, and nature, has been linked to an increased likelihood of clinically significant depressive symptoms. Interestingly, chronic stressors have been associated with a lower probability of advanced disease, while in PDAC patients, more severe acute life events correlate with worsening depressive symptoms over time [74]. These findings suggest a complex interplay between stress exposure, psychological distress, and disease progression in PC.

Interleukin-6 is a cytokine that has a proven effect on the pathogenesis of PC [75] and depression [76]. The IL6 gene encodes this protein, and macrophages and T-cells secrete it. Its activity includes pro- and anti-inflammatory effects, and it serves as a mediator of stress-induced immunosuppression. IL6 has been linked to the induction of cancer cell stemness, which can lead to tumorigenesis, and it can also promote cancer cell metastasis by downregulating E-cadherin [77]. The impact of IL6 on the development of depression manifests itself in the downregulation of brain-derived neurotrophic factor levels [78,79]. It can also mediate the modified connectivity of the anterior cingulate cortex [78]. Noradrenaline can generate the production of IL-6 in pancreatic cancer cells [26]. The available studies measured IL6 levels at 0, 1, 4, 8, and 24 h after noradrenaline administration. Higher IL6 secretion was observed after 4 h and continued to increase to 80 times the normal level after 24 h of noradrenaline administration. That development could be repressed by the admission of sulforaphane [26].

It has been established that patients with depression have altered metabolism of the prefrontal lobe, which leads to flawed activity of key neural circuits [80]. That change in the operation of the brain can be a source of mood disorders in patients with cancer. A study was also performed that measured cerebral glucose metabolism rates between patients with PC and depression and patients with PC without depression [81]. It has been revealed that in patients with depression, cerebral glucose metabolism rates were much higher in the subgenual anterior cingulate cortex than in the control group. That region is highly associated with the presence of mood disorders [82]. There were no major differences between these two groups in other brain regions.

Although direct evidence of central neural circuit alterations in pancreatic cancer patients is lacking, PC has been reported to have one of the highest incidence rates of depression among other tumors [83]. Moreover, depression can also precede PC diagnosis, suggesting tumor-driven biological mechanisms rather than solely a psychological reaction to illness. In a large US population study of 62,450 pancreatic cancer patients, up to 21% experienced depression prior to cancer diagnosis [84]. The proposed mechanism involves overexpression of indoleamine 2,3-dioxygenase (IDO), an enzyme that induces metabolic changes implicated in depression. In PC, IDO overexpression is stimulated by pro-inflammatory cytokines, mainly interferon-γ. This redirects tryptophan metabolism from the serotonin pathway toward the kynurenine pathway, reducing serotonin availability and increasing neuroactive metabolites’ production [85]. Overactivation of the Kyn pathway leads to an imbalance in neurotoxic/neuroprotective compounds, which may contribute to depressive symptoms [86]. Although depression does not appear to affect overall survival in PC, it markedly worsens QOL and psychological well-being, underscoring the need for early recognition and management [87].

#### 3.2.2. Ovarian Cancer

Ovarian cancer (OC) is diagnosed 225,000 times each year worldwide, which makes it the seventh most common cancer in women [88]. It is also a type of cancer characterized by a low survival rate, with only 35% of affected women surviving 10 years after diagnosis [89,90]. As for such a serious disease, we have little knowledge of how chronic stress affects the course of the OC disease. Women diagnosed with gynecological cancers, particularly those with early-stage borderline ovarian tumors, experience significant physical and psychological morbidity beyond that observed in other cancer populations. Evidence indicates a higher prevalence of anxiety, stress, and sexual dysfunction in these patients compared to controls, despite similar demographic characteristics. These findings emphasize the importance of systematic screening and psychosocial support addressing anxiety, depression, and sexual health during postoperative follow-up [91]. Studies show that chronic, but not acute, stress exposure is associated with higher levels of depression, worse sleep quality, and reduced overall well-being during the first year after diagnosis in OC patients. These findings emphasize the importance of assessing long-term stressors and implementing stress-reduction interventions as part of comprehensive care for OC patients [92]. Women with gynecological cancer frequently experience sleep disturbances following treatment, influenced by factors such as self-stigma, perceived stress, and cultural or social pressures. Research indicates that higher perceived stress significantly increases the likelihood of developing moderate to severe sleep disorders, while self-stigma indirectly contributes to these problems through its effect on stress. These findings highlight the need for psychosocial interventions aimed at reducing self-stigma and anxiety to improve sleep quality and overall well-being in women with gynecological cancers [93]. Reassuringly, among women with newly diagnosed epithelial OC, the first six months of survivorship are marked by significant improvements in both physical and emotional QOL, as well as a reduction in perceived stress. These positive changes occur regardless of disease stage or treatment approach, suggesting that modern therapeutic strategies do not necessarily impair patient-reported outcomes. The findings highlight the importance of monitoring psychological recovery early in survivorship to support sustained well-being in OC patients [94].

OC represents a significant interpersonal stressor, with patients’ QOL strongly influenced by the dynamics within their intimate relationships. Insecure attachment styles were associated with poorer social and functional quality of life, largely due to reduced engagement in common dyadic coping strategies. Although shared coping can have positive effects, it may also become emotionally exhausting, underscoring the complex role of relational factors in the psychological adjustment of women facing ovarian cancer [95].

As we turn to the molecular level, among neurotransmitters, serotonin has been examined as a stress-related transmitter that might influence the development of OC [96]. Serotonin plays several roles in the human body; among other things, it regulates mood and emotions, controls the sleep/wake cycle, and has an important role in oocyte maturation [97]. It was exhibited that using selective serotonin reuptake inhibitors reduces the risk of the most common type of ovarian cancer, which is epithelial OC [98]. Such a revelation suggests that serotonin may be linked to OC development. The effect of serotonin on cancer cells depends on the type of receptors the cells express. In one of the studies, SK-OV-3 cells that can form tumors with a histology similar to that of human OC were used [99]. DNA sequencing analysis revealed that the presence of the most common serotonin receptor in a healthy ovary, 5-Hydroxytryptamine Receptor 1E (HTR1E), is significantly lowered in OC tissue [100]. The level of HTR1E expression was also examined in 20 patients with OC and peritoneal dissemination. RT-PCR analyses were conducted, which confirmed lower levels of HTR1E expression in cancer cells [96]. To confirm that this receptor has suppressive activity against the ovarian tumor, SK-OV-3 cells were implanted into mice. In one group of the animals, cancer cells were unaltered; however, in the second group, levels of HTR1E receptors on cancer cells were significantly lowered. Mice with a lower level of HTR1E receptor in cancer cells presented faster tumor growth and had a higher number of metastases in the peritoneal cavity [96]. In addition, SK-OV-3 cells were cultured in a medium containing 0.5 μM of serotonin, which showed that the growth of cells with the HTR1E receptor was slower than that of HTR1E receptor-free cells. Increasing the serotonin concentration in the medium to 5.0 μM further inhibited the growth of HTR1E-positive cells, but no significant effect on HTR1E-negative cells was observed. That shows that serotonin can inhibit the proliferation of cancer cells [96]. However, serotonin levels in the body can vary depending on chronic stress [101]. Lower serum serotonin levels, from which ovarian serotonin comes, were detected in mice subjected to chronic stress [102]. It shows that chronic stress can affect serotonin presence in the body negatively, which in turn can influence the proliferation of cancer cells and increase the possibility of forming metastases [96].

In several studies, the correlation between VEGF levels and social support in patients with OC was examined [66]. Angiogenesis is essential for the tumor to reach significant measures. The maximum diameter achieved for most tumors without developing blood vessels is around 1–2 mm [103]. Thus, when the cancer reaches these measurements, it starts producing substances that allow the growth of blood vessels, which enables the tumor to achieve a significant size and form metastases [103,104]. One of the described substances is VEGF, the secretion of which is regulated by several factors, including IL-6, gonadotropic hormones, and noradrenaline [105]. It has been proven that IL-6 levels are increased with acute and chronic stress [106]. That leads to the assumption that increased levels of IL-6 during stress can increase the secretion of VEGF by the tumor and consequently promote angiogenesis and further cancer development (Figure 5b).

### 3.3. Therapy and Chronic Stress

Cancer is a traumatic disease which, for many patients, leads to feelings of anxiety, depression, fatigue, or hopelessness [107,108]. Psychotherapy aims to alleviate stress-related biological responses, which may indirectly enhance therapeutic efficacy by improving the patient’s physiological resilience. Nevertheless, medicine has developed effective ways to improve patients’ well-being. Pharmacotherapy is possible; however, it is often connected with a high amount of side effects [109]. Among the nonpharmacological approaches, mindfulness-based cognitive therapy plays a significant role as supportive therapy [110]. It aims to disengage patients from unhealthy beliefs, emotions, and thoughts and simultaneously enforce their emotional well-being. Studies have proven that practices of this kind effectively lower anxiety and depression in patients [110]. In BC patients, reductions in fatigue and improvements in overall QOL were also observed [111]. However, it seems that such therapy is more suited to women, as they respond better to the treatment [110]. The presented situation has two reasons. Mainly men are not keen on sharing emotions during group sessions, which can significantly hinder their progress [112]. The second reason is that men at the start exhibit lower levels of anxiety, which results in worse improvements throughout therapy [113].

Recent findings underscore the critical link between chronic stress and adverse outcomes in cancer patients, including heightened symptom severity, suffering, and mortality. This connection elevates psychological care to a cornerstone of comprehensive cancer treatment, aiming to enhance patient QOL [114]. Psychotherapy allows patients to gain a new perspective on their current situation, which can help them pursue positive goals and create a feeling of hope [115]. Such therapies provide a supportive environment in which patients can freely express their worries and negative emotions, thereby positively contributing to their overall well-being and potentially impacting their treatment journey [116]. The study by Vitale et al. effectively used the Kessler-10 (K10) scale in a population of cancer patients to assess the effectiveness of psychotherapy. It was observed that psychotherapy played a significant role in alleviating this distress within months of commencement. Interestingly, the authors observed differential impacts of psychotherapy across educational and employment status groups, highlighting the need for personalized psychological support strategies [117]. The other study analyzed cancer patients with high levels of emotional distress, which was enhanced by psychological support, resulting in reduced posttraumatic stress [118]. However, several factors can affect the overall therapeutic effect. It was noted that patients responded better to hospital or other health center sessions than to those held in the community [119,120]. This can result in hospital personnel, including nurses, being more qualified to care for oncological patients [121]. Another factor that can affect the effects of psychotherapy is whether the patient participates in sessions alone or with a partner or caregivers. One study shows that better results are achieved when therapy is conducted alone, without group discussion [115]. Patients become more willing to self-explore and have the message more tailored to their individual needs while working with the therapist alone. The most significant enhancement in hopefulness throughout psychotherapy was notably observed in patients diagnosed with breast cancer (BC). This phenomenon may be attributed to the predisposition of women to discuss their psychological distress more readily compared to men. Additionally, women typically report lower initial levels of hope at the commencement of therapy, which consequently leads to more pronounced improvements in hopefulness as treatment progresses [115]. Another factor that could increase the effectiveness of therapy is assigning homework, which would help patients integrate their sessions into their daily lives [122]. Interestingly, a randomized controlled trial was proposed using the eHealth application for cancer patients to determine mental health within an 8-week program. This application included Mindfulness-Based Cognitive Therapy for Cancer (MBCT-Ca), Positive Psychology (PP), and Autogenic Training (AT)—and was delivered to breast cancer survivors post-adjuvant treatment. Assessing both psychological and biological stress markers, the trial aimed to elucidate the impact of the interventions on heart-rate variability, depression, anxiety, stress, QOL, and positive mental health, as well as secondary outcomes such as serum cortisol levels, immunomarkers, sleep quality, and fatigue. Utilizing a combined model repeated measures approach, this study stands out by examining the differential effects of tailored eHealth interventions on mental health indicators and their potential to improve stress biomarkers, thereby contributing to better recovery and overall health in cancer patients [123].

Another well-established therapeutic approach in the psychological care of BC patients is Cognitive Behavioral Stress Management (CBSM). In a randomized controlled trial among women after surgery for BC, CBSM has been found to decrease emotional distress, intrusive thoughts, and anxiety [124]. It has also been reported to reduce the prevalence of moderate depression [125], lower serum cortisol levels [126], and downregulate the expression of pro-inflammatory and metastasis-related genes in peripheral leukocytes, particularly NF-κB expression [125,126]. A long-term follow-up trial in non-metastatic breast cancer further found that CBSM participants exhibited lower all-cause and breast cancer–specific mortality. However, the authors advised cautious interpretation of these findings [127]. Sleep-focused interventions may also attenuate inflammatory activity. In a randomized study of breast cancer survivors, both Cognitive Behavioral Therapy for Insomnia (CBT-I) and Tai Chi reduced circulating inflammatory biomarkers, including CRP and IL-6 [128]. Exercise interventions represent another promising avenue for reducing inflammation. In a randomized trial among colorectal cancer survivors, 12 weeks of moderate-intensity aerobic training resulted in a reduction in hs-CRP and IL-6 levels in stage III patients [129]. Similar anti-inflammatory effects have been observed in breast and prostate cancer patients [130,131].

Even though therapy can positively affect patients, sustaining this effect is not easy. The effects on hope, anxiety, emotional distress, and depression were all reported to decline after the end of the sessions [132]. A significant increase in patients’ negative emotions could be seen as soon as one month after finishing sessions. The situation significantly worsened after three months of finishing therapy [115]. For psychotherapy to be truly effective, we have to find methods to make it last longer. What is more, it could be an innovative approach to begin psychotherapy in patients from the risk groups rather than after the neoplasm is already diagnosed. Thus, increasing social awareness of the benefits of psychological support not only in healthcare-related issues might decrease the scale of negative feelings that arise once a patient is diagnosed with a neoplastic disease. It could even lower their incidence by reducing stress and promoting healthy life patterns.

### 3.4. Clinical Implications

The interplay between chronic stress and cancer progression not only underscores the necessity for routine psychological evaluations in oncological care but also highlights the potential therapeutic benefits of integrating psychotherapeutic interventions into standard cancer treatment regimens. Clinically, addressing psychological distress through tailored psychotherapy may help reduce the overall physiological burden associated with chronic stress responses; however, these interventions act within a broader network of biological and environmental determinants. For cancers with traditionally low survival rates, such as pancreatic and ovarian cancer, psychotherapeutic interventions could be particularly beneficial in improving outcomes by reducing stress-induced tumorigenesis and enhancing patient resilience. Conversely, in cancers with higher survival rates, like breast and prostate cancer, psychotherapy could support maintaining a positive outlook, which has been associated with better treatment responses and lower morbidity. Healthcare systems should consider adopting standardized psycho-oncological support, not only to enhance patient QOL but also as a potentially cost-effective strategy to improve clinical outcomes. This approach requires collaboration across oncology and mental health disciplines, ensuring that psychological care is not an adjunct but an integral part of cancer care protocols.

Previous studies often discuss stress and cancer either broadly across malignancies or within a single cancer type. Our review reveals evidence using an explicit survival stratification and then compares cancer type-specific stress responsivity and mechanisms across breast and prostate cancers versus pancreatic and ovarian cancers. This structure creates two direct benefits for researchers and clinicians: it separates pathways most consistently linked to invasion, metastasis, and therapy resistance from those dominated by inflammatory and cytokine-driven biology, and it aligns these mechanisms with evidence on psychosocial interventions to identify testable, risk-adapted psycho-oncologic strategies.

## 4. Conclusions

This systematic review demonstrates a significant association between chronic stress and cancer progression. While persistent activation of the HPA axis and sympathetic nervous system is linked to inflammation and immunosuppression, future research must aim to disentangle these multifactorial biological and environmental influences to determine causal directionality. In contrast, psychotherapeutic and stress-management interventions can alleviate stress-related biological responses, improving patients’ well-being and potentially enhancing therapeutic efficacy. These findings emphasize the importance of integrating psycho-oncological support into standard cancer care to improve clinical outcomes and QOL. Associations between Quality of Life (QOL) and mortality should be interpreted with caution, as reduced QOL may reflect advancing disease severity rather than a direct independent driver of mortality. Future research should aim to disentangle the multifactorial influences, psychological, biological, and environmental, that collectively modulate hormone and cytokine regulation in cancer.

## Data Availability

No new data were created or analyzed in this study. Data sharing is not applicable to this article.
